# In the Information Age, do dementia caregivers get the information they need? Semi-structured interviews to determine informal caregivers’ education needs, barriers, and preferences

**DOI:** 10.1186/s12877-016-0338-7

**Published:** 2016-09-23

**Authors:** Kendra Peterson, Howard Hahn, Amber J. Lee, Catherine A. Madison, Alireza Atri

**Affiliations:** 1 Ray Dolby Brain Health Center; California Pacific Medical Center, a Sutter Health Affiliate, San Francisco, CA USA; 2 California Pacific Medical Center Research Institute, San Francisco, CA USA; 3 Geisel School of Medicine, Dartmouth College, Hanover, NH USA; 4 Brigham and Women’s Hospital and Harvard Medical School, Boston, MA USA; 5 45 Castro Street, Suite 220, San Francisco, California 94114 USA

**Keywords:** Caregiver education, Caregivers, Dementia, Information, Barriers

## Abstract

**Background:**

Most patients with dementia or cognitive impairment receive care from family members, often untrained for this challenging role. Caregivers may not access publicly available caregiving information, and caregiver education programs are not widely implemented clinically. Prior large surveys yielded broad quantitative understanding of caregiver information needs, but do not illuminate the in-depth, rich, and nuanced caregiver perspectives that can be gleaned using qualitative methodology.

**Methods:**

We aimed to understand perspectives about information sources, barriers and preferences, through semi-structured interviews with 27 caregivers. Content analysis identified important themes.

**Results:**

We interviewed 19 women, 8 men; mean age 58.5 years; most adult children (15) or spouses (8) of the care recipient. Dementia symptoms often developed insidiously, with delayed disease acknowledgement and caregiver self-identification. While memory loss was common, behavioral symptoms were most troublesome, often initially unrecognized as disease indicators. Emerging themes: 1.) Barriers to seeking information often result from knowledge gaps, rather than reluctance to assume the caregiver role; 2.) Most caregivers currently receive insufficient information. Caregivers are open to many information sources, settings, and technologies, including referrals to other healthcare professionals, print material, and community and internet resources, but expect the primary care provider (PCP) to recommend, endorse, and guide them to specific sources.

**Conclusions:**

These findings replicated and expanded on results from previous quantitative surveys and, importantly, revealed a previously unrecognized essential factor: despite receiving insufficient information, caregivers place critical value on their relationship with care recipient PCPs to receive recommendations, guidance and endorsement to sources of caregiving information. Implications include: 1.) Greater public education is needed to help caregivers identify and describe diverse cognitive, functional and behavioral symptoms that lead to dementia, and recognize the benefits of early detection in accessing information regarding multi-modality management and care; 2.) Improved methods are needed for PCPs to detect and manage cognitive and behavioral changes, as well as mechanisms that facilitate the busy PCP, either directly or via referral, to provide caregiver information, education, support, and services. The critical relationship between caregivers and PCPs should not be circumvented but should be facilitated to provide more effective guidance regarding dementia caregiver needs.

**Electronic supplementary material:**

The online version of this article (doi:10.1186/s12877-016-0338-7) contains supplementary material, which is available to authorized users.

## Background

There are currently over 46 million people living with dementia worldwide [1]. In the United States there are 5.4 million people living with Alzheimer’s Disease [2], and more individuals are affected when other forms of dementia or cognitive impairment are included [3, 4]. With an aging demographic, these numbers are expected to dramatically rise in coming decades. More than 80 % of people with dementia are cared for at home by one or more informal caregivers, usually unpaid family members or friends [2, 5, 6]. Although not all identify with the term caregiver, we will use this term as shorthand in this report. More than 15 million are estimated to be informal caregivers of people with dementia in the U.S. [2, 5, 7]. Because there is often a delay from the time of symptom onset until diagnosis [7], additional caregivers are likely to be providing care to those who do not yet have an established diagnosis.

The role of caregiver to a person with dementia or cognitive impairment (referred to hereafter as care recipient) often levies enormous costs to time, emotional well-being, physical health, and finances [8, 9]. Although some caregivers demonstrate resilience and coping strategies that help ameliorate a negative experience [10, 11], for many it is an extremely challenging role, and one for which most caregivers have little background or training [7]. More than 80 % of caregivers report needing more information on caregiving topics [12].

Most caregivers turn to a primary care provider (PCP) for information, but the information obtained there is often limited [5, 13–15]. Randomized clinical trials of more than 200 psychoeducational interventions support efficacy in improving knowledge and support of dementia caregivers [16]. Caregiver education and support programs improve caregiver confidence; reduce caregiver depression, distress, and upsetting thoughts; improve life satisfaction and response to disruptive behaviors; reduce behavioral and psychological symptoms; and delay nursing home placement of care recipients [17–21]. However, few such programs are widely implemented outside a research setting. Additionally, in the Information Age [22] a vast amount of beneficial information is publicly available through disease-specific and caregiving organizations, but many caregivers do not access that information [7].

Multidisciplinary specialty memory/dementia clinics provide comprehensive care that includes extensive caregiver support and education [23]. Yet, most patients with dementia receive the bulk of their medical care in primary care settings [24, 25], where resources of busy PCPs to offer comprehensive services and caregiver education are limited.

In this study we sought to better understand the complex determinants that lead informal caregivers of people with cognitive impairment or dementia to recognize their need for caregiving education. Further, we aimed to assess caregivers’ sources for information, barriers to seeking and receiving information, and preferences for information sources. Although the subject of information sources for dementia caregivers has been examined previously in large quantitative surveys [7, 12], we aimed to explore in depth using qualitative semi-structured interviews caregivers’ perspectives, impressions, and opinions, in order to develop a more rich and nuanced view of this subject.

## Methods

### Design

We collected data from participants using semi-structured interviews (SSI), and used the qualitative method of directed content analysis [26], to explore with each caregiver participant (CG) their thoughts regarding a set of previously determined caregiving-related interview topics. We then explored in depth areas that emerged as being of importance for individual participants.

### Participants

Per an IRB-approved protocol, a single investigator (KP) identified, screened, contacted, obtained verbal consent, and interviewed CG participants. Unlike the other investigator’s KP does not function in our Center in a clinical capacity to evaluate or treat patients, and has no interactions with patients or caregivers. Having only KP identify, screen, contact, obtain consent, and interview CG participants ensured anonymity and confidentiality for the participants should they accompany the CR to the clinic in the future.

CGs were identified via a prospective sequential search of our specialty memory/dementia clinic schedule for upcoming initial/new patient evaluations, between August and November of 2015. Electronic health records were then screened to locate documentation of: 1) an identified caregiver or confidante for the patient, with a contact telephone number; and either, 2) a Referring Diagnosis of memory or cognitive issues; or, 3) a Problem List diagnosis of memory or cognitive issues; or, 4) a Medication List prescription for a dementia-specific medication.

Telephone contact was attempted for 77 eligible caregivers; 47 were reached by telephone, and 27 CG completed interviews. Using an IRB-approved standard script, the project’s purpose and the voluntary nature of participation were explained, and participants were advised that they would not be identified in any future communications or publications. Verbal consent was obtained for participation; the IRB waived the requirement for written informed consent due to low risk of participation. All caregivers contacted were provided Caregiver Education Resources (cpmc.org/brainhealth then go to “Cognitive Symptoms & Caregiver Strategies”).

### Data collection

An Interview Guide (Additional file 1), with questions on pre-determined topics (Table 1), was developed prior to participant interviews, via literature review and investigator consensus. After collection of demographic data, the CR’s functional severity was estimated by KP, based on the description provided by the CG. Functional severity was categorized into: mild impairment (CR requires some support including with reminders about events, appointments, or medications or has mild behavioral changes, but is otherwise carrying out majority of activities of daily living independently); moderate impairment (CR required substantial assistance with ADLs including organizing affairs, managing money, and travel); and, severe impairment (CR required full care for most ADLs and had trouble recognizing people, trouble communicating, and/or severe behavioral disturbances).

**Table 1:** Interview Topics (See Additional file 1 for Interview Guide)

ᅟ	ᅟ
1) Choice in becoming a Caregiverᅟ	
2) Duration of Care Recipient Symptoms	
a) Prior to Interview	
b) Prior to medical evaluation	
3) Care Recipient Symptoms	
a) Initial	
b) Most troubling	
4) Information regarding diagnosis and treatment (Triggers, previous sources, most helpful sources, barriers, expectations and preferences)	
5) Information regarding care issues and strategies (Triggers, identification as caregiver, previous sources, most helpful sources, barriers, expectations and preferences)	
6) Preferred learning methods and settings	
7) Use of Technology	
a) Internet	
b) Computer applications	
c) Touch screen learning device	

Participants were then asked the same series of open-ended questions regarding pre-determined topics. Follow-up questions were tailored to the individual participant’s responses, with the purpose of clarifying and expanding on areas revealed to be of most interest or concern to the participant. Detailed participant interview notes, including representative verbatim quotes, were taken during the interviews. The interviews were not taped, thus further ensuring participant anonymity and confidentiality, as the investigators who would participate in the clinical care of the CRs when they came to the clinic did not have the opportunity to hear the voices of participants, or to read specific details in a verbatim transcript, that may potentially lead to later recognizing them if they were to meet in person. The interview notes were grouped by topic as a mechanism for organizing the material, and transcribed.

### Data analysis

Transcripts were analyzed by three investigators (KP, HH, AL) using the technique of directed content analysis. Transcribed notes were reviewed to identify and code common themes (specific phenomena), identified by common words and similar explicit or inferred meaning that was contained within the responses. Coded themes were not predetermined, but were guided by identification of specific phenomena that emerged during review of the transcripts. Themes that arose during the interviews were quantified in order to detect the frequency with which they occurred during interviews. Frequency of emerging themes was considered an indicator of their importance, and is therefore presented in Tables 4 and 5.

Representative quotations were derived from individual unidentified participants. More than one participant may have responded with content similar to that quoted, but the quotation was included when it was considered to be illustrative of a particular point.

## Results

### Caregiver participant (CG) characteristics

Demographics and characteristics of CGs are shown in Table 2. CGs were mostly women (70 %), had an average age of 58.5 years, were all fluent in English, resided in the San Francisco Bay Area, and were mostly adult children (55.2 %) or spouses (30.0 %) of CRs.

**Table 2:** Caregiver Characteristics [*n* = 27]

Age	
mean years (SD)	58.5 (12.2)
median years (range)	59 (34–86)
Gender	*n* (%)
Female	19 (70 %)
Male	8 (30 %)
Educational Background	
High school only	7 (25.9 %)
Some college	5 (18.5 %)
College graduate	11 (40.8 %)
Post-graduate degree	4 (14.8 %)
Language	
Fluent in English	27 (100 %)
English second language	3 (11.1 %)
Residing SF Bay Area	27 (100 %)
Relationship of CG to CR	
Adult Child	15 (55.6 %)
Daughter	10 (37 %)
Daughter-in-law	1 (3.7 %)
Son	4 (14.5 %)
Spouse	8 (30 %)
Wife	7 (26.3 %)
Husband	1 (3.7 %)
Other relative	2 (7.4 %)
Niece	1 (3.7 %)
Grandson	1 (3.7 %)
Friend	2 (7.4 %)
Principle Caregiver	27 (100 %)
Sole Caregiver	13 (48.1 %)
Another Caregiver	14 (51.9 %)
Another family member	11 (40.7 %)
Paid caregiver	3 (11.1 %)

### Care recipient (CR) characteristics

Demographics and characteristics of CRs are shown in Table 3. CRs were mostly women (55.6 %), had an average age of 79.8 years, and mostly had mild or moderate estimated functional dependence (88.9 %).

**Table 3:** Care Recipient (CR) Characteristics [*n* = 27]

Age	
Mean years (SD)	79.8 (9.2)
Median years (range)	83 (57–92)
Gender	*n* (%)
Female	15 (55.6 %)
Male	12 (44.4 %)
Diagnosis (as understood by CG)	
Unknown	14 (51.9 %)
Dementia, nonspecific	6 (22.2 %)
Alzheimer’s Disease (AD)	3 (11.1 %)
Stroke-related (VCI)	2 (7.4 %)
Dementia with Lewy Bodies (DLB)	1 (3.7 %)
Parkinson’s Disease with Dementia	1 (3.7 %)
Estimated Functional Severity (see text for definitions)	
Mild	9 (33.3 %)
Moderate	15 (55.6 %)
Severe	3 (11.1 %)

### Reponses to interview topics

#### Choice in being in the caregiving role

All CGs were asked if they had a choice in caring for their loved one. Twenty-four CGs responded affirmatively that they were caring for their loved one willingly, giving reasons such as “life is a partnership”, “I want to take care of her”, “it’s my role in the family”, or “she has been like a mother to me”. Three CG expressed ambivalence, stating they did it somewhat willingly but that there was no one else to do it, so in that sense they “did not have a choice in the matter”, it was an “obligation” or “both choice and necessity”. None expressed outright resentment or anger about being in the caregiving role.

#### Duration of care recipient symptoms

CGs estimated the duration of CR symptoms prior to the interview. Estimates ranged from a few months to 12 years; however, many found it difficult to determine precisely when symptoms began. Twenty-three noted insidious onset, describing symptoms that “snuck up on them”; thus, they also assumed caregiving tasks gradually.

The duration of CR symptoms prior to seeking medical advice was also difficult to determine; estimates ranged from “right away” to several years. Three reported the CR sought medical advice within a month of symptom onset (2 had stroke and 1 had post-operative cognitive changes). The rest reported that, in retrospect, symptoms were present for months (13 CG), or months-to-years, before seeking medical advice.

#### CR Symptoms

CGs described the initial symptoms, and symptoms that were most troubling or difficult to manage (Table 4). The most common initial symptom was memory loss: the CR repeated himself, forgot names, appointments, conversations, dates, facts, or medications. The most troubling symptoms related to personality, mood, and behavior changes: depression, loss of interest in activities, agitation, anger, frustration, stubbornness, inflexibility, bluntness, rudeness, paranoia, or childishness. Five CG volunteered that the CR became more agitated and disoriented in the evening, or throughout the night with disrupted sleep.

**Table 4:** Care Recipient Symptoms

Initial Symptoms [*n* = 36*]	*n* (%)
Memory Loss	23 (63.9 %)
Personality/Mood/Behavior Change	5 (13.9 %)
Executive Functions	4 (11.1 %)
Acute/subacute confusion/cognitive impairments	4 (11.1 %)
Most Troubling Symptoms (for Caregiver) [*n* = 34**]	
Personality/Mood/Behavior Change	20 (58.8 %)
Memory Loss	9 (26.5 %)
Safety or getting lost	5 (14.7 %)

*some caregivers reported more than one initial symptom

**some caregivers reported more than most troubling symptom

Executive dysfunction, with problems multi-tasking, planning, reasoning and processing complex information, translated into difficulties with activities such as following recipes or tying shoes. Safety issues included getting lost, leaving the stove on or the door unlocked; visual processing impairment led to a car accident for one CR.

All CGs recognized memory problems as a possible sign of dementia or “senility”. But 13 CGs volunteered that they were initially unaware that personality, mood, and behavioral changes were potential indicators of a medical illness, and early on had not considered that there could be treatments or supportive interventions to help alleviate these symptoms.

#### Distinguishing Medical Information from Caregiving Information

We intended to distinguished information specific to medical diagnosis and treatment, from information regarding symptom management and caregiver strategies, as we explored triggers, sources, barriers, and expectations (Table 1, Topics 4 and 5). However, this was not a construct that most CGs had considered. Eighteen CG did not distinguish these two types of information and expected both to come together from the same sources; 8 reported they were not previously aware that any information about caregiving would exist.

However, once the distinction between medical and caregiving information was clarified, CG were asked about the specific kinds of information they would be interested in learning; 5 of 9 CG of mildly affected CRs mostly wanted on information about diagnosis and medical treatments, (“What is wrong and how can we fix it?”) and were less interested in learning to manage symptoms. For CRs with moderate or severe functional impairments, often later in the disease course with a known diagnosis, 13 of 18 CGs expressed interest in both medical and caregiving information.

#### Triggers for Seeking Medical Evaluation and Caregiver Information

CGs were asked if a specific trigger or threshold moment led to the CR’s medical evaluation or seeking caregiving information. Seventeen could not identify such a trigger, stating that in retrospect they were vaguely aware that “something wasn’t right” for months or years before a gradual accumulation of symptoms finally prompted medical evaluation and information seeking. The other 9 CGs identified triggers including an abrupt memory or cognitive decline, problematic behavioral symptoms, declining functional capabilities, or increased CG frustration. One CR with a strong family history of Alzheimer’s Disease but only mild memory symptoms sought medical evaluation very early.

CGs were also asked about the evolution in their role, and their identification with the term caregiver. For most, the role evolved gradually. It was often difficult to determine precisely when the balance in the relationship had shifted from simply a caring family member or friend, to one who was actually providing care to a loved one who could no longer function independently. Despite acknowledging their role in assisting a family member who had cognitive impairment, 15 had not self-identified with the term caregiver prior to the interview. Although 12 reported that they did apply the term caregiver to themselves, 9 said that in retrospect they realized that they had functioned as a caregiver for “a while” before it occurred to them that they were in the role. Only 3, of whom 2 had prior professional health care experience and 1 who had been referred early to a support group, reported they had self-identified as a caregiver early in the course of assuming the role.

#### Previous sources of information

CGs were asked about prior sources of medical or caregiving information. Six reported they had not yet sought or received any medical or caregiving information, and were waiting to receive information at their scheduled appointment. Of these, one had prior professional experience, and another had actively resisted accessing any of the sources of information previously recommended by another specialty clinic.

The other 21 CG reported they had initiated the CR medical evaluation and sought information from a prior physician, in most cases a PCP (2 from other specialty clinics). CG variably used the term PCP, primary doctor, doctor, or primary physician, and since we did not specifically distinguish the type of provider, we use the term PCP to include primary care physicians as well other primary health care providers, such as nurse practitioners. But 17 reported that the PCP had done little evaluation, and that despite asking, they had received very little or no information from the PCP. No CG reported that the CR’s cognitive issues were first brought to their attention by a PCP, or that the PCP offered unsolicited information. CG stated that the PCP “didn’t notice the symptoms, even though I had been noticing for years”, “minimized the symptoms” or “attributed the symptoms to aging”, or that when brought to the PCPs attention he “didn’t evaluate much but just made the referral to the specialty clinic.” Only 4 CG reported that a prior physician had provided useful information or referred them to allied health professionals, a support group, or written information sources.

Eleven CG had searched the Internet for information regarding the CR’s illness. However, many found the Internet experience frustrating and unrewarding in providing targeted information. An exception was one CG who spent copious time on the Internet reading and forwarding many sources to the CR’s physician.

Four CG reported they had received advice and information from others in their communities who had encountered similar experiences, but found it difficult to assess the relevance or reliability of the information provided, especially if the CR’s diagnosis was not yet known. One CG had attended a helpful seminar at a Senior Center, while another had read two books on caregiving.

#### Most helpful sources

As a group, CGs perceived that they had received little information about the disease, treatment, or caregiving, and no single most helpful and reliable information source emerged. For many there was a gap between the current desire for information and what CGs had so far received.

#### Barriers to receiving information

CGs were asked to identify barriers that had kept them from getting information earlier. They identified both their own barriers to seeking information, and barriers to the information sources. (Table 5).

**Table 5:** Barriers to Receiving Information

Barriers to CG Seeking Information [*n* = 59]*	*n* (%)
CG did not initially acknowledge symptoms as a disease (esp. behavioral)	24 (40.7 %)
CG did not know the diagnosis or what terms to search for	11 (18.6 %)
CG did not know there would be information available (esp. behavioral)	10 (16.9 %)
CG too busy or overwhelmed to seek information	6 (10.2 %)
CG in denial or avoiding stigma of disease	4 (6.8 %)
CG didn’t think of self as “caregiver”	3 (5.1 %)
CG already familiar professionally	1 (1.7 %)
Barriers to Information Sources, [*n* = 38]	*n* (%)
Insufficient information provided by PCP	17 (44.7 %)
PCP too busy to provide information	5 (13.2 %)
Internet search frustrating	16 (42.1 %)

*CG* caregiver, *PCP* primary care provider

*some CG reported more than one barrier

#### Barriers to information seeking

Twenty-four CGs described that initially they had been uncertain whether the changes observed in the CRs were something to be concerned about, and had thought the changes were part of normal aging or, when behavioral changes were prominent, that the care recipient was “just being difficult”. One CG explained, “If he’d had a fever, I would have known what to tell the doctor. But at first when he just acted more belligerent than he used to, I wouldn’t have known what to call that, and I didn’t even think about it being a medical problem that I should bring up with the doctor.”

Even when a medical problem was suspected, 11 CG reported that until a specific name of a disease or diagnosis was mentioned, they did not know how to go about searching for or obtaining information. Ten CGs reported they had been unaware that there was information available regarding caregiving, especially strategies to alleviate problematic behavioral symptoms, and felt resigned that “there was nothing to be done”. They viewed their situation as uncommon or unique, were unaware that many other caregivers have faced similar problems, and that a body of knowledge existed to assist caregivers. A common refrain was that a CG was “simply doing the best I can and figuring it out as I go”.

Due to delay in seeking information, some CGs expressed they had reached a point of feeling “desperate” or “at their wit’s end” prior to seeking medical or caregiving advice. Six CGs indicated they were too busy and overwhelmed, both from the tasks of caregiving and from managing other aspects of their lives (children, work, other commitments) to devote time to learning more about the disease, treatments, or caregiving strategies. They had not considered that investing some time learning strategies to help manage symptoms might eventually lead to time saving and reduced frustration. Four CGs said they had actively resisted seeking medical advice or information, stating that they had been “in denial, “hoping that it would just not get any worse”, or “avoiding the stigma of the disease”. Others described not wanting “to embarrass” the CR or to “rock the boat” in the relationship.

One CG with professional knowledge regarding dementia and caregiving had not sought additional information when faced with the situation in her own family. However, she acknowledged that when assuming the role of a caregiver to a family member the experience was different, and more challenging, than anticipated.

#### Barriers to information sources

Seventeen CGs identified as a barrier that the PCP had provided little or no information. In most cases the CG perceived that the PCP did not actively screen or assess the CR for cognitive issues, even when it was brought to the PCP’s attention by the CR or CG, and seemed to minimize the significance. This suggested to the CG that there was nothing to be done (despite several PCPs prescribing dementia-specific medications). Often, no diagnosis was established or discussed, and this led many CGs to feel as if they did not have “the right words to talk about the problem”, did not have the necessary vocabulary to communicate with the PCP or to seek information on their own.

Five CGs volunteered that the PCP was too busy to discuss complex problems or provide education. One CG reported he educated the physician instead, by providing him with detailed information from the Internet.

Sixteen CGs reported inefficiency of Internet searches as a barrier; those who had used the Internet for this problem reported feeling overwhelmed by the hundreds or thousands of references they found, and had no reliable mechanism to filter search results for relevancy and accuracy. Many described being unsure if they could “trust” Internet sources, and not know which sites were “good ones” to look at; a few relied on strategies such as turning to “.org” or “.gov” sites, or to sites of specific medical institutions that they considered reliable.

Most CGs searched for information regarding the medical diagnosis by using general terms such as “memory loss”, or “dementia”, if that term had been used by the PCP. They were much less likely to be aware of, or to look for, sites that were devoted to caregiving issues. Few were able to cite a specific Internet site that they had found particularly helpful. Unless they had been specifically told a diagnosis of Alzheimer’s Disease, they were unlikely to search for sites related to this term; only 5 reported visiting sites of nationally recognized disease-specific or caregiving organizations. While a few CGs found the Internet to be extremely helpful, the most common emerging theme was that the process of searching the Internet was time consuming, frustrating, and futile.

#### Expectations and preferences for information

CGs were asked from what sources they would expect or prefer to receive medical and caregiving information.

#### PCPs as crucial trusted portal for information and referrals

Despite generally reporting that they had not received much information from the PCP about cognitive and caregiving issues, CGs still expressed the expectation that the principal source of health information is a physician; the PCP would be the “port of entry” into them receiving necessary information and would facilitate this process. They generally indicated confidence in PCPs and “put a lot of stock in what the doctor says”. Many expressed disappointment that the PCP had not spent more time assessing the CR’s cognitive issues and providing helpful information. However, CGs were also sympathetic to demands of busy physicians’ schedules, and some indicated that PCPs could not be expected to have an in-depth understanding of every medical problem. So while CGs would have preferred to receive more information directly from the PCP, they also accepted some limitations.

Twenty-two CGs volunteered their expectation that if the PCP was unable to provide this information himself, he would recommend and endorse other specific sources including: referrals to reputable specialists or allied health professionals (e.g. nurse, case manager, social worker); pamphlets/written materials; and appropriate classes, support groups, and Internet sites.

#### Preferred sources and settings for learning

CGs were asked about their preferred sources and settings for learning health information. The most important factor mentioned by 22 CGs was that the physician recommends the information source and setting. They considered the physician’s endorsement to be an indication of the credibility and quality of the information they would receive. No other dominant preferred source emerged; CGs were open to receive information in several modalities including 1:1 personal interaction and teaching by a physician or allied health professional; an interactive classroom experience; written materials (which allowed them processing smalls bits of information and returning to information source as needed); and video, audio, or slide presentation. There was also no dominant preference for learning setting; these included the physicians’ office, classroom, and own home with printed materials or Internet sources. CGs with English as a second language (primary language: Spanish 2, Cantonese 1) preferred written or verbal instruction in their primary language, though they reported being able to learn the information adequately if presented in English.

#### Technology

CGs were asked to report their familiarity and use of various forms of technology.

#### Internet

All CGs reported access and use of the Internet for some purposes. Eight volunteered that they use the Internet to search for general health-related information, and 7 stated they do not. Eleven stated they had tried to use the Internet for information regarding the CR’s issues, but most could not identify sites they regularly used, and reported they often search for general terms such as “memory loss”. Most CGs reported wanting to be directed to reputable and relevant sites by a trusted health care provider.

#### Computer/mobile applications

CGs were asked if they were familiar with or used desktop or mobile applications (apps); 23 reported being familiar or using them to some degree. When asked if they were likely to use a computer or mobile app, if available, to assist them in caregiving tasks, only five reported they would; two elaborated they would if recommended by a physician.

#### Touch screen learning device

CGs were presented with the concept of accessing health information in general, and specifically information about cognitive issues and caregiving, on a touch screen learning device. It was proposed that such a device might become available in the waiting room of their physician’s office, in other health care settings such as pharmacy or hospital lobby, or in other non-medical public settings. The CGs would use the device to select from a menu of topics those they were interested in learning about, then would be provided with the information they selected in print or by email.

Most CGs responded positively and with interest to this concept; 17 reported if they encountered such a device while waiting in a physician’s office, they were “very likely” to engage with it, while 8 reported they “might” use it. The location of the device was crucial. Twenty-five reported that if they encountered the device in a physician’s waiting room they would view its information as “reliable” or “endorsed by” the physician, while if the device were placed in a more general health care setting outside an individual physician’s waiting room/office they were less likely to view the information as reliable. Eight volunteered that a physician’s specific recommendation would make it even more likely that they would use it, as it would enhance the perception that the information contained was educational rather than “advertising”. All CGs, except one, reported that they would not be likely to engage with such a device if encountered in a public, non-medical setting.

Other factors mentioned by CGs that would affect the likelihood of engaging with such a device included the amount of time spent in the waiting room where the device was located, and the device’s ease of use.

Major themes that emerged in regard to the interview topics, along with the implications that we derived from these themes, are presented in Table 6.

**Table 6:** Themes and Implications

Emerging Themes	Implications of Emerging Themes
CG would not choose for their loved ones to be ill, but many take on the role of caregiving willingly when needed.	A willing caregiver can be enlisted by the PCP as an active partner in the patient’s care.
There is often a long delay between symptom onset and diagnosis, and a corresponding delay in CG self-identifying their role. Memory loss is the most common initial symptom, but behavioral symptoms are often most troublesome, and often not recognized to indicate disease.	Improving CG ability to recognize and describe cognitive and behavioral symptoms, and their role as “caregiver” earlier, could prompt earlier diagnosis and information. This could be approached through public education, and PCP screening for both cognitive and behavioral symptoms.
Many CG do not distinguish medical diagnosis and treatment from symptom management and supportive care.	Effective caregiver education includes both medical and caregiving information.
Many CG attempt to obtain information from a PCP or the Internet, but get little information and are frustrated by Internet searches. CG are open to a wide variety of sources, settings, and technologies, but rely on the PCP to provide information and guide them to other sources.	Better mechanisms are needed to facilitate busy PCPs to provide information and referrals to other specific sources, including reliable Internet sites.

## Discussion

Our study used SSI with content analysis to reveal several prominent themes related to CG perceptions and perspectives about information sources, barriers and preferences, as summarized in Table 6. While several of these themes have previously emerged in broad quantitative surveys, an important novel theme emerged: despite endorsing not having received adequate information from PCPs, caregivers continue to place critical value on their relationship with care recipient PCPs to receive recommendations, guidance and endorsement to sources of caregiving information in the early stages of the caregiving journey.

A first theme that emerged was that *while CGs would not choose for their loved ones to be ill*, *many caregivers choose to take on the role of caregiving when needed*. On the surface, this finding appears to be at odds with the lack of choice reported by about half of caregivers in an AARP-NAC survey [12]. However, this may reflect a difference in semantics arising from the different methodologies used to assess choice of caregiving; compared to survey methodology, the SSI methodology enabled us to clarify the CGs’ interpretation of the word choice. Our findings do not support a hypothesis that most caregivers do not seek information because they are resistant to being in the role. An important implication of our findings is that many caregivers could be enlisted by health care providers as active care partners in patient care.

A second set of themes that emerged was that *there is often a long delay between the insidious onset of symptoms and the caregiver’s acknowledgment of disease*, *and thus a corresponding delay in a caregiver identifying with the role as a caregiver*. Our findings also support those of others [27–30] that *while memory loss is commonly the initial symptom noticed*, *behavioral and personality changes are more commonly the most troublesome and difficult symptoms to manage*. Importantly, our results reveal that while many caregivers recognize the potential implications of progressive memory loss for a possible dementia diagnosis, they initially may not recognize that behavioral symptoms could indicate a medical disease, and, furthermore, appear unaware of the existence of non-pharmacological strategies available to help with these symptoms.

A potential confound to recognizing, and measuring, clinically significant cognitive decline due to dementing conditions is the problem of appropriately considering baseline level of premorbid intellectual function, or cognitive reserve: the individual differences in how people process tasks allow some to cope better than others with brain pathology [31, 32]. Conceptually, individuals with higher cognitive reserve show more resilience to, and can better functionally compensate for, multiple forms of neurologic insult, as well as depression [33]; they also manifest slower rates of decline in normal cognitive aging, and can have delayed onset, but faster apparent decline in AD/dementia [31]. Our study did not consider the potential confound of cognitive reserve on the dynamics of caregiver recognition of clinical effects of disease and their own need for education; it is plausible that these would both be delayed in cases when care recipients have higher reserve.

We also found that *caregivers often lack the vocabulary to describe symptoms to the PCP or to search for information themselves*, especially if a diagnosis has not yet been established or discussed. However, CGs in our study reported that with progression of symptoms, they eventually alerted the PCP, rather than the PCP first detecting and assessing the problem.

Important implications of these findings are that improving caregivers’ ability to recognize and describe both cognitive and behavioral symptoms, as well as acknowledging their own role as caregiver, is likely to prompt earlier diagnosis and earlier provision of caregiver information. Public education campaigns could give caregivers a more effective vocabulary to report symptoms to PCPs. Furthermore, effective screening by the PCP for cognitive symptoms as part of the Medicare Annual Wellness Visit [34], and importantly, also effectively screening for behavioral symptoms, could prompt earlier diagnosis and earlier provision of vital information.

Another important theme that emerged was that *caregivers hold a relatively holistic view of medical diagnosis*/*treatment and behavioral strategies*/*caregiver support*. While health care providers may conceptualize and organize information to distinguish medical diagnosis and treatment from symptom management and supportive care, this may not be a meaningful or helpful distinction to most caregivers. Though physicians may focus on medical diagnosis and treatment, leaving the latter to other allied health providers (e.g. nursing, social work, palliative care), our results reveal that this was not a paradigm that makes sense to caregivers, who expect guidance about both a disease and caregiving strategies to come from the same sources, preferably initiated by the PCP. This implies that effective caregiver education should include early access to both medical and caregiving information.

The final crucial theme that emerged is that *despite CGs reporting that they have received little information from PCPs or from other publically available sources*, *they endorse a strong trust in*, *and reliance on the PCP as the first source of health and caregiving information*. CGs were sympathetic in their assessment that PCPs may not have the knowledge or time to provide all of this information themselves, but still expected the PCP to be their port of entry into reliable health information, and to make specific recommendations to direct them to the information they need. While CGs were open to learning caregiving information from different sources in a variety of settings and technologies, no dominant source, setting, or technology emerged as being crucial for all CG, and it may be that a variety of sources is necessary to reach different caregivers. The most important factor to validate use of available information was for their PCP to make a specific recommendation, referral or endorsement. The implication of this finding is that there is vital necessity for implementing better methods to facilitate detection and management of clinically relevant cognitive and behavioral changes in the PCP office (e.g. by providing and incentivizing specific dementia-related education, remuneration and resources) such that PCPs can either directly provide accurate diagnostic, treatment, education and caregiver support themselves, or can refer patients and caregivers to other reputable sources. Optimally, both would occur, with the PCP beginning the process, and then reinforcing and managing it in collaboration with other referrals and resources.

Our study has several important strengths, in addition to some limitations. A primary strength of the study is the advantageous and novel application of rigorous SSI and content analysis techniques to assess our specific study aims. Several previous qualitative studies focusing on a wide range of CG experiences have been reported [13–15, 35–40]. But to our knowledge, this is the first study using SSI methods to specifically focus on the perceptions and issues of information needs, barriers, and preferences for dementia caregivers. Unlike large population surveys containing data on caregiver information sources reported previously, SSI allowed in-depth and nuanced explorations that gained a rich narrative of data from a relatively modest sample of caregiver subjects regarding their perceptions and impressions, thus enriching the understanding of the rationale and motivations underlying caregiver responses. Going beyond the general pre-determined topic questions, follow-up interview questions tailored to individual subject responses allowed us to obtain both a broader (and more sensitive) and deeper (more specific and precise) understanding of the topics. Although we quantified and reported the incidence of emerging themes among the interviews, a limitation of using SSI for data collection is that the quantification may not be as precise as would be expected with a structured survey using specific pre-determined questions. Nonetheless, we considered the frequency of emerging themes arising in the interviews to be an indication of their relative importance, and therefore included this data in our report.

A possible limitation of our study is that the interviews were not recorded, although the interviewer took extensive written notes during the interview, including representative verbatim quotes. However, this approach did ensure anonymity of the participants and confidentiality of the interview content, since the investigators who would later see the CR and CG for clinical purposes would not be able to recognize the voices from a taped interview or potential specific identifying content from a verbatim transcript. Although we acknowledge that this method of data collection may have resulted in incomplete data retention, in designing the study we balanced the potential limitation in data rigor and precision in favor of the strength of ensuring full anonymity of the participants and confidentiality of their responses.

Another study strength is that it mirrored caregiver demographic characteristics of previous large-population surveys of caregivers in the United States [2, 5, 7, 12], and is likely to be representative of caregiver perspectives, particularly those caregivers living in urban areas of the U.S. While, unknown factors that prompted referral of these CRs to our urban specialty memory clinic might potentially distinguish our group of CGs from those in the general U.S. population, the CGs in our study include representative subjects of both genders, a wide range of ages, educational backgrounds, and durations of CR symptoms. A potential limitation of our study, that may underestimate the relative magnitude of barriers to caregivers receiving information, is that we did not specifically query the ethnic and racial characteristics of our subjects. All CGs were fluent in spoken English (and reported being fluent in written English), but for minority English-non-fluent CGs not included in the study the identified barriers are predicted to be even more pronounced. Similar to prior population surveys, our CGs were relatively well-educated, but the most salient barriers are likely to have an even greater impact on less educated caregivers. For example, the top three barriers to seeking information (see Table 5) were responsible for over 75 % of the identified barriers – all these barriers are knowledge/education-based barriers.

While CGs in our study were, on average, college-educated and about 59 years old, 42 % of the major causes for information source barriers identified were due to Internet searches and sources being frustrating or unreliable, (45 % were due to PCPs not providing sufficient information), and 82 % of CGs reported wanting the PCP to be the primary source of information and referral. These issues may be even more present in caregivers with less education and facility with the Internet, and in those who place greater reliance on their PCP.

Another potential limitation, that may limit generalization to a much broader population of silent caregivers who are at earlier stages of the dementia evaluation process or caregiving journey, is possible bias in CG selection. The CGs in our study were already down a path of referral to our single specialty memory clinic; as such their perspectives may not fully represent those of caregivers not referred to our clinic or caregivers even earlier in the process, or even their own perceptions during a previous stage in their caregiving journey – though they were asked about this, their responses required retrospective thought about their prior state. Also, while CGs were interviewed in a systematic way for this study, the CRs they described were not directly examined by study staff for the purposes of this study, so the determination of functional severity reported was an estimate based on the description by the CG; however, even in more formal classification schemes, functional severity is typically determined via caregiver report. Finally, of 77 potential participants identified by screening, we completed interviews for only 27, thus raising the possibility that the participants we interviewed and their responses were not fully representative of the entire group.

Despite wide availability of information on a plethora of caregiving topics, most CG in our study still expected the PCP to be the first source of medical and caregiving information and referral. However, there remains a disparity between expectation and actual experience; our results support findings from prior surveys in which only a third of caregivers report that a health care provider had inquired what was needed to care for the care recipient; even fewer caregivers are reportedly asked about self-care [7, 12].

Our results further illuminate the critical value caregivers place on their relationship with care recipient PCPs. CGs were open to a range of information sources including referral to allied health professionals or medical specialists, written materials, support groups, classes, Internet sites, or a variety of other technological solutions, but they want the PCP to recommend and endorse specific sources. These findings provide further support that caregivers want and could benefit from receiving education and information about dementia diagnosis and treatments (including prognosis and expectations for treatments), and for learning strategies to address issues that commonly arise when providing care; issues such as behavioral changes, communication, daily activities, advance care planning, respite, and self-care. Providing multifactorial approaches to early detection, management, psychoeducation, care and support for patient-caregiver dyads is associated with better long-term outcomes in Alzheimer’s disease [41–45].

Current dementia caregivers are predominantly older; thus it is possible that the ways caregivers seek and receive information will evolve in coming years as a younger generation, who has grown up using technology even more extensively, become caregivers. Future caregivers may prove to be more facile and reliant on technological information sources than is the current generation. However, it is our contention that the important human element of medicine is unlikely to change, and caregivers’ and patients’ trust and reliance on the PCP as the first authority and arbitrator on health issues will endure. Furthermore, with an increasing number of dementia patients and a shortage of dementia specialists available to diagnose and treat them, PCPs will continue to have a prominent role in their diagnosis, treatment, and providing education and support for their caregivers [7, 24, 46].

## Conclusions

The most prominent recurring themes regarding caregiver perceptions, barriers and preferences emerging from this study are: 1.) The top barriers to caregivers seeking information are due to caregiver knowledge gaps (as opposed to reluctance to assume the caregiver role); and, 2.) While caregivers are open to receive information in a variety of ways, they found the information they obtained (via PCP, internet, etc.) insufficient, and have a strong expectation that the PCP should recommend, endorse, and guide them to specific sources of care and information, which can include referrals to other healthcare professionals, print material, and community and internet resources. Implications of these findings further support that: 1.) Greater public education is needed regarding the diversity and spectrum of symptoms of cognitive, functional and behavioral changes that lead to dementia, and regarding the benefits of early detection, and multi-factorial management and care; and 2.) Improved methods are needed to implement better detection and management of cognitive and behavioral changes in the PCP office (e.g. by providing and incentivizing specific dementia-related education, remuneration and resources) and to facilitate provision (either directly or via referral) of caregiver information, education, support, and services. Fostering a more effective relationship between caregivers and care recipient PCPs remains critical to providing crucial early guidance regarding support and education resources for caregivers.

## Additional file

Additional file 1: Interview Guide. Semi-Structured Interview questions (developed for this study; not previously published.). (DOCX 20 kb)

## Abbreviations

CG: Caregiver participant (in our study)
CR: Care recipient
PCP: Primary care provider
SSI: Semi-structured interview
