# Supplementary material for: In the Information Age, do dementia caregivers get the information they need? Semi-structured interviews to determine informal caregivers’ education needs, barriers, and preferences
Source: BMC Geriatr. 2016 Sep 23;16:164. doi: 10.1186/s12877-016-0338-7 (PMC5035467; doi:10.1186/s12877-016-0338-7)
Supplement: Additional file 1: — Interview Guide. Semi-Structured Interview questions (developed for this study; not previously published.). (DOCX 20 kb) [file 12877_2016_338_MOESM1_ESM.docx]

INTERVIEW GUIDE

Background Information:

CG Age

CG Gender

CG Highest Level of Education

CG Language

Residence

Relationship to Care Recipient

CR Age

CR Gender

CR diagnosis, if known

Duration CR had symptoms prior to interview

CR functional severity: (Estimate based on CG description of CR and needs for assistance.)

Semi-Structured Interview Questions:

What was the first symptom(s) that you noticed?

What was the most troubling symptom(s) to you?

How long did CR have symptoms before s/he saw a doctor for the symptoms?

How long has CR needed your help?

Is this the first person you have helped take care of with a similar condition?

Do you consider that you had a choice in helping to take care of CR?

Does CR have other people who help take care? Are there paid caregivers?

Have you received any information about the disease and medical treatment options?

If so, from what sources? (Explore: Doctor, other medical provider, print materials from doctors office, family, friends, other people taking care of someone with the same condition, library, other print materials, internet (what sites), support groups, classes)

If so, what sources of information have you found to be most helpful? Why?

What lead you to receive information? Was that information that you asked for or went looking for, or did someone offer it to you without your asking?

If you haven’t received information, do you know why not?

Have you received information about the tasks involved in helping take care of someone with cognitive problems?

If so, from what sources? (Explore sources as above)

If so, what sources of information have you found to be most helpful?

What made you recognize that you needed additional information in order to help take care of CR? Do you identify yourself with the term caregiver?

Are there any places or people that you would expect to receive this kind of information from, but haven’t?

Are there any places or people that you would prefer to receive this information from?

What methods do you prefer to learn from? (Explore: Directly from another person, one on one; with a teacher in a class; from a support group of others facing the same issues; written materials; Internet or other digital sources; slide show; audio program; video.)

What settings do you prefer to learn this kind of information in? (Explore: at home; in a classroom or other group setting; in a public or health library; in a doctor’s clinical exam room; in a doctor’s waiting room.)

Do you use the Internet? Would you know how to search for information about caregiving topics on the Internet? What search terms might you use, or what sites might you go to?

Do you know what computer or telephone applications or apps are? Do you use any? If an app were available to help take care of CR, how likely do you think it is that you would use it?

If you were to see a touch screen in a doctor’s office (similar to a TV screen, or like an automated kiosk that you use at a bank) that had a menu of educational information on it that you could select to have emailed to you, do you think you would approach and use the device? Would the setting (waiting rooms cafeteria, hospital lobby, or in other public space) affect the likelihood of your using?
